# Malaria control in South Africa 2000–2010: beyond MDG6

**DOI:** 10.1186/1475-2875-11-294

**Published:** 2012-08-22

**Authors:** Devanand Moonasar, Tej Nuthulaganti, Philip S Kruger, Aaron Mabuza, Eric S Rasiswi, Frew G Benson, Rajendra Maharaj

**Affiliations:** 1 National Department of Health- South Africa, Civitas Building, Cnr Andries & Struben Street, Pretoria, Gauteng, 0001, South Africa; 2 Harvard School of Public Health, 677 Huntington Avenue, Boston, MA, 02115, USA; 3 Clinton Health Access Initiative and Global Health Group, University of California, San Francisco, Pretoria, South Africa; 4 Limpopo Provincial Government: Department of Health and Social Welfare, PO Box 33, Tzaneen, 0850, South Africa; 5 Mpumalanga Provincial Government: Department of Health and Social Services, Private Bag X11278, Nelspruit, Mpumalanga, South Africa; 6 KwaZulu-Natal Provincial Government: Department of Health– KwaZulu-Natal, Private Bag X002, Jozini, KwaZulu-Natal, South Africa, 3969; 7 Medical Research Council of South Africa, Malaria Research Programme, Medical Research Council, PO Box 70380, Overport, Durban, 4067, South Africa

**Keywords:** Malaria elimination, South Africa, Vector control, Case management and Millennium Development Goals

## Abstract

**Background:**

Malaria is one of the key targets within Goal 6 of the Millennium Development Goals (MDGs), whereby the disease needs to be halted and reversed by the year 2015. Several other international targets have been set, however the MDGs are universally accepted, hence it is the focus of this manuscript.

**Methods:**

An assessment was undertaken to determine the progress South Africa has made against the malaria target of MDG Goal 6. Data were analyzed for the period 2000 until 2010 and verified after municipal boundary changes in some of South Africa’s districts and subsequent to verifying actual residence of malaria positive cases.

**Results:**

South Africa has made significant progress in controlling malaria transmission over the past decade; malaria cases declined by 89.41% (63663 in 2000 vs 6741 in 2010) and deaths decreased by 85.4% (453 vs 66) in the year 2000 compared to the year 2010. Coupled with this, malaria cases among children under five years of age have also declined by 93% (6791 in 2000 vs 451 in 2010). This has resulted in South Africa achieving and exceeding the malaria target of the MDGs. A series of interventions have attributed to this decrease, these include: drug policy change from monotherapy to artemisinin combination therapy, insecticide change from pyrethroids back to DDT; cross border collaboration (South Africa with Mozambique and Swaziland through the Lubombo Spatial Development Initiative– LSDI) and financial investment in malaria control. The KwaZulu-Natal Province has seen the largest reduction in malaria cases and deaths (99.1% cases- 41786 vs 380; and 98.5% deaths 340 vs 5), when comparing the year 2000 with 2010. The Limpopo Province recorded the lowest reduction in malaria cases compared to the other malaria endemic provinces (56.1% reduction- 9487 vs 4174; when comparing 2000 to 2010).

**Conclusions:**

South Africa is well positioned to move beyond the malaria target of the MDGs and progress towards elimination. However, in addition to its existing interventions, the country will need to sustain its financing for malaria control and support programmed reorientation towards elimination and scale up active surveillance coupled with treatment at the community level. Moreover cross-border malaria collaboration needs to be sustained and scaled up to prevent the re-introduction of malaria into the country.

## Background

South Africa has three malaria-endemic provinces: Limpopo, Mpumalanga and KwaZulu-Natal. Ninety-five percent of all malaria infections in South Africa are due to the parasite species *Plasmodium falciparum* and the local vector is predominantly *Anopheles arabiensis*[1]. Malaria is transmitted mainly during the rainy season from October to May and transmission peaks normally in January and April. In South Africa, an estimated 10% of the population are living in malaria-endemic areas and are at risk of contracting the disease. Malaria transmission occurs mainly in the northern (bordering Zimbabwe) and eastern (bordering Mozambique) parts of the country. The South African malaria programme dates back to the early 1940s, when key WHO-recommended strategies have been employed to control the disease [2].

South Africa has been very active in contributing to malaria prevention and control policies in southern Africa, the African continent, and at the global level. South Africa has committed to a series of international declarations on malaria, including the United Nations Millennium Development Goals (MDGs) [3,4]. The MDGs were launched in 2000 during the United Nations Millennium Declaration and were adopted in 2001. The MDGs comprise of key goals to alleviate poverty and disease and improve human development. Several governments, non-governmental and multilateral agencies use the MDGs (eight in total) as a universal yardstick to monitor individual countries' progress on health performance. Goal 6 of the MDGs (MDG6) was set to combat HIV/AIDS, malaria and other diseases and has the target of halting these diseases by 2015 and to begin to reverse the incidence of malaria and other infectious diseases.

In this paper, the authors review the progress South Africa has made in achieving the malaria-related MDGs and highlight factors that have contributed to reducing malaria cases and deaths in South Africa. In addition, the authors also share useful lessons and identify priority interventions that South Africa needs to adopt and sustain to go beyond Goal 6 of the malaria-related MDGs.

Malaria is related to MDGs 4, 5 and 6, data for MDG 4 and 5 are associated with other programmatic areas and were determined to be beyond the scope of this manuscript. This paper focuses mainly on the malaria target of MDG 6 as data was most comprehensively available to assess South Africa’s performance against this goal.

Data were obtained from the malaria information systems at the national Department of Health in the South Africa, where data are routinely reported. Outcomes were measured by evaluating malaria cases and deaths over the period 2000 to 2010. Data from 2000 were established as the baseline year corresponding to the launch of the MDGs. GIS maps were produced to highlight the origin of malaria cases as reported by patients seen at health facilities, thereby enabling the identification of transmission foci.

## Methods

### Strategies for malaria control

South Africa has been implementing evidence-based malaria control interventions in keeping with the WHO’s strategy for malaria control. The key strategies include: surveillance, vector control, health promotion, case management, and cross-border malaria initiatives.

### Surveillance strategy

Malaria is a notifiable disease in South Africa since 1956 [5]. All malaria cases are therefore by law required to be notified to health authorities in the country. Effective malaria information systems have been developed in the three malaria-endemic provinces of South Africa, with the support of the South African Medical Research Council and implemented since 1997 [6]. A malaria case is defined as a person who is *Plasmodium* positive by slide microscopy or through a rapid diagnostic test (RDT) [7]. Ideally notification data should be collected and analyzed through the District Health Information System, however due to the challenge of slow data entry and the need to track outbreaks of malaria cases, the Malaria Information System (MIS) is used.

The MIS is a useful adjunct to the District Health Information System, especially for the malaria-endemic provinces. Malaria case data is routinely collected by malaria control staff and entered into the MIS at each of the provincial malaria programmes in the endemic provinces. In addition to the passive reporting system, active case detection of index cases and screening populations in close proximity to these cases are being conducted in two of the three malaria-endemic provinces: Mpumalanga and KwaZulu-Natal; with this activity being limited in the Limpopo Province.

### Vector control strategy

South Africa has been implementing Indoor Residual Spraying (IRS) coupled with focal larviciding for malaria vector control since the 1930s [8]. Large-scale larviciding using Paris Green was discontinued from the malaria programme as it was labor intensive, requiring weekly treatments. IRS intervention has proved relatively successful since the first trials were conducted by Hamilton and Ross using Pyagra (liquid pyrethrum and kerosene) in Kwazulu-Natal in 1932 and has since been the mainstay of the malaria control programme in the country [9].

Dichlorodiphenyltrichloroethane (DDT), an effective insecticide for malaria control, replaced the use of Pyagra in 1946 in KwaZulu-Natal and other parts of South Africa and complete coverage was achieved in 1958 [9]. The use of DDT for IRS was phased out of the malaria control programme in Mpumalanga and KwaZulu-Natal provinces in 1996 due to negative community perceptions over the use of the chemical, and was replaced with synthetic pyrethroids. Deltamethrin was proven at the time to be an equally effective chemical for malaria vector control. In 2000, Hargreaves *et al.* reported pyrethroid-resistant *Anopheles funestus* (which was previously eradicated) in Northern KwaZulu-Natal; between 11% and 50% of those mosquitoes were resistant to permethrin (14–25% in first generation offspring), with the sporozoite rate being 5.4% from collected individuals [10]. This sparked concern from malaria control authorities, and after widespread consultation (including members of a National Malaria Advisory Group- consisting of malaria experts and programme staff which was set up in 1994) it was decided by the national Department of Health to change the chemical for malaria vector control back to DDT, which showed 100% mortality for collected mosquitoes. DDT, deltamethrin and carbamates remain the chemicals of choice for IRS in South Africa [1].

### Health promotion strategy

Health promotion has been a key strategy in the South African malaria programme for several decades. Health promotion is used to influence community behavior towards the preventative and curative components of malaria control strategies. On the preventative front, the strategy is used to ensure that communities comply with instructions from spray operators during IRS campaigns and take personal, protective measures against being bitten by malaria-infected mosquitoes [11]. On the curative side, health promotion strategies are used to help communities recognize the signs and symptoms of malaria and seek early treatment. Information, education and communication (IEC) messaging is delivered through several channels: mass community events, radio talk shows and during sport matches. Health messaging is delivered through public personalities including: sports players and musicians.

### Case management strategy

South Africa employs definitive diagnosis for malaria case confirmation and treatment. Malaria diagnosis and treatment is provided free of charge in public sector facilities. The country has been at the forefront of revising policy to ensure effective drugs are used in its malaria control program. The selection of drugs was based on sound scientific evidence. Historically, South Africa used chloroquine in its malaria program to treat uncomplicated malaria and quinine for complicated malaria. Drug resistance to chloroquine was first reported in KwaZulu-Natal in 1987, where Freese *et al.* in an *in vitro,* drug resistance study found 88% parasite resistance [12]. Drug policy subsequently changed from chloroquine to sulphadoxine-pyrimethamine (SP) in 1988, in KwaZulu-Natal. Chloroquine resistance was also reported in Mpumalanga and Limpopo Provinces, necessitating drug policy change to SP in 1997 [13,14].

SP resistance started to increase in the mid-1990s and reached approximately 80% by 2000, requiring a change in drug policy to Coartem® in 2001, in KwaZulu-Natal [15]. In 2004, Limpopo Province replaced SP with Coartem® and Mpumalanga Province used SP-artesunate in the public sector from 2001 to the end of 2005 and has implemented Coartem® since January 2006 [7].

### Cross-border malaria strategy

Since malaria is mainly transmitted along its northern and eastern borders, South Africa has been collaborating on malaria control with its neighboring countries. The two initiatives that have been established in the past decade are: the Trans-Limpopo Malaria Initiative (TLMI) and the Lubombo Spatial Development Initiative (LSDI) [2].

The TLMI is aimed at achieving reduction in malaria transmission in the Matabeleland South Province (Beitbridge municipality) of Zimbabwe and the Limpopo Province (Musina Municipality). Its key strategy has been to ensure policy harmonization and synchronization of malaria interventions such as case management, vector control, surveillance and health promotion across bordering districts of the two countries.

The LSDI is a joint programme between the governments of Mozambique, Swaziland and South Africa to develop the Lubombo region of eastern Swaziland, southern Mozambique (Maputo Province) and north-eastern KwaZulu-Natal into a globally competitive economic zone [16]. The rationale for the LSDI was that the Lubombo regions consist of poor communities that are affected by malaria, hence tackling malaria in this region would increase tourism, thus resulting in economic development in these areas. Furthermore, through a down-streaming effect, reduction of malaria would result in a decrease in transmission in the border areas of South Africa. The key interventions of the LSDI were: vector control through IRS and parasite control through first-line treatment with artemisinin combination therapy (ACT).

## Results

**Figure 1:**
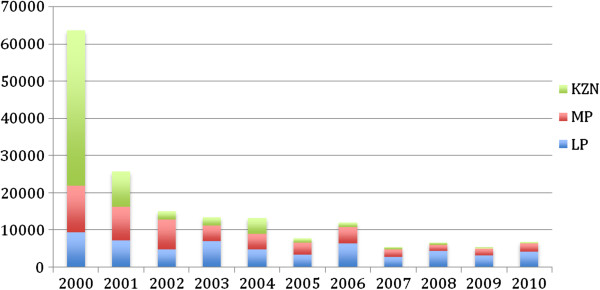
**Malaria cases from the year 2000 to 2010 in South Africa’s malaria endemic provinces: Limpopo, Mpumalanga and KwaZulu-Natal.** Data was obtained from the national routine reporting information system.

South Africa has made significant progress in controlling malaria since 2000. The total malaria cases in South Africa declined by 89.41% in 2010 compared to the baseline year 2000 (63663 to 6741 cases respectively)(see Table 1). Malaria deaths have also declined by 85.4% for the same comparison years (453 to 66 deaths in 2000 and 2010, respectively). Since the baseline year in 2000, malaria cases decreased in 2001 by 59.6% (63663 to 25731 cases) and experienced further reductions in subsequent years (Figure 1). Similarly, malaria deaths rapidly declined in 2001 by 74.8% (453 to 114 deaths) compared to 2000 and deaths were reduced further in following years.

**Table 1:** Total malaria cases and deaths, stratified by province and municipality for the endemic regions of South Africa for the year 2010 vs 2000

**Province**	**District*(municipality)***	**Malaria**		**Decrease in malaria cases n (%)**	**Malaria Deaths**		**Decrease in malaria Deaths n(%)**
**Years**		**2000**	**2010**		**2000**	**2010**	
**National**	**-**	63663	6741	56922 (89.41%)	453	66	387 (85.4%)
**Limpopo Province**	-	9487	4174	5313 (56.1%)	68	40	28 (41.1%)
	***Vhembe***	5453	2558	2895 (53.1%)	51	20	31 (60.0%)
	***Mopani***	2627	1368	1259 (48.0%)	19	13	6 (31.6%)
**Mpumalanga**	**-**	12390	2187	10203 (82.3%)	45	26	19 (42.2%)
	***Nkomasi***	10398	1120	9278 (89.2%)	33	7	26 (78.7%)
	***Bushbuckridge***	801	559	242 (30.2%)	4	11	7 -*Increase* (63.4%)
**KwaZulu- Natal**	**-**	41786	380	41506 (99.1%)	340	5	335 (98.5%)
	***Umkanyakude***	38543	85	38458 (99.7%)	84	3	81 (96.4%)
	***Uthungulu***	1941	53	1888 (97.2%)	32	2	30 (93.75%)
	***Zululand***	686	6	680 (99.1%)	4	1	3 (75.0%)

**Figure 2:**
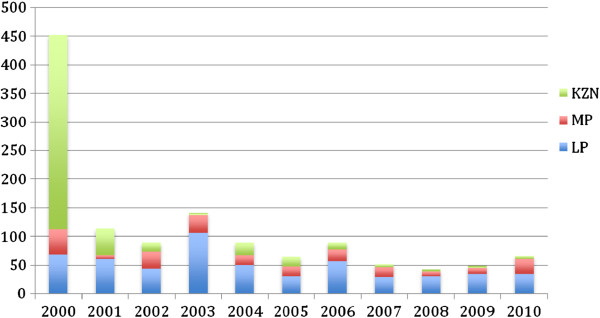
**Total malaria deaths from the year 2000 to 2010 in South Africa’s malaria endemic provinces: Limpopo, Mpumalanga and KwaZulu-Natal.** Data was obtained from the national routine reporting information system.

However, in 2006 malaria cases and deaths increased in the endemic provinces of South Africa, mainly in Limpopo and Mpumalanga Provinces. Cases remain below 7000 for the period 2007 to 2010 with malaria deaths fluctuating: ranging from 51 to 66 for the same period (Figure 2). The key reason for this increase was due to importation of malaria cases from neighboring endemic countries, with resulting secondary transmission.

In 2000, KwaZulu-Natal Province reported the highest burden of malaria of all endemic provinces (65.6% of total malaria burden in South Africa). Kwazulu-Natal is the province that has subsequently experienced huge reductions in malaria cases, after initial reduction in 2001. Cases over this period declined by over 77% (41786 cases in 2000 to 9473 cases in 2001). Subsequently in KwaZulu-Natal, malaria cases have been steadily decreasing, by 99.1% in 2010 compared to the baseline in 2000 (41786 *vs* 380 cases). Malaria deaths followed a similar downward trend in KwaZulu-Natal for the same comparison years, with 99.1% reduction (Table 1; Figures 1 and 2).

In Mpumalanga Province malaria cases have steadily declined by 82.3% in 2010 compared to baseline year 2000 (12,390 *vs* 2187). Malaria deaths have also been reduced in Mpumalanga Province by 41.2% (45 deaths in 2000 compared to 26 deaths in 2010) for the same comparison years.

Malaria cases have also been reduced by 56.1% in Limpopo Province (9487 *vs* 4174 cases) between 2000 and 2010.

When data were stratified to the district levels, malaria cases and deaths had consistently reduced across all the malaria-endemic districts. The KwaZulu-Natal districts of Umkanyakude, Utungulu and Zululand saw, on average, a reduction of malaria cases of approximately 99%. The percentage reduction in deaths in KwaZulu-Natal followed a similar downward trend in each of the districts with the reductions ranging from 75.0% to 96.4%.

Whilst malaria cases in Bushbuckridge decreased over the reporting period, malaria deaths increased. This stems mainly from late presentations to health facilities. Malaria cases in the Nkomasi district of Mpumalanga Province reduced by 89.1% in 2010 compared to 2000 levels, with a 78.7% reduction in deaths for the same comparison years.

Malaria case reduction has also been recorded in the Vhembe and Mopani districts of the Limpopo Province with declines of 53.1% and 48.0% for 2010 *vs* 2000, with corresponding reduction in deaths of 60% and 31% in the respective districts for the same comparison years.

In South Africa’s three endemic provinces, the total malaria cases for children under five decreased by over 93% (9513 total cases in 2000 compared to 603 total cases in 2010 – see Table 2). The reduction in the number of cases and deaths due to malaria has been consistent throughout the endemic districts among children under five years of age.

**Table 2:** Malaria cases and deaths stratified by under 5’s for the endemic regions of South Africa for the year 2010 vs 2000

**Province**	**District*(municipality)***	**Malaria cases in under 5's**	**malaria cases in under 5’s**	**Reduction in under 5 cases n (%)**	**Malaria deaths in under 5's**		**Reduction in malaria deaths in under 5’s n (%)**
**Years**		**2000**	**2010**		**2000**	**2010**	
**National**	**-**	6791	451	6340 (93.4%)	14	1	13(92.8%)
**Limpopo Province**		854	275	579 (68.0%)	3	0	3 (100.0%)
	***Vhembe***	527	173	354 (67.1%)	1	0	1 (100%)
	***Mopani***	275	90	185 (67.2%)	2	0	2 (100%)
**Mpumalanga**	**-**	1467	233	1234 (84.1%)	1	1	0 -
	***Nkomazi***	825	137	688 (83.4%)	1	0	1 (100%)
	**Bushbuckridge**	57	37	20 (35.1%)	0	0	0 -
**KwaZulu- Natal**		5284	22	5262 (99.5%)	4	1	3 (75.0%)
	**Umkanyakude**	4925	10	4915 (99.8%)	9	1	8 (88.8%)
	**Uthungulu**	184	3	181 (98.3%)	0	0	0 -
	**Zululand**	55	0	55 (100%)	1	0	1 (100%)

## Discussion

The period of 2000–2010 was chosen for the study as data for this period was verified subsequent to municipal boundary changes over those years in South Africa and residential verification of positive cases was also available. Verified data for subsequent years were not available at the time of the study. In addition, this study focused mainly on data from endemic provinces, as this data-set was comprehensive for the analysis period and only the total cases recorded at the health facility were recorded and analyzed.

South Africa has significantly reduced its malaria burden over the decade 2000–2010, hence the country has now embraced a program of malaria elimination (zero local transmission of cases) targeting elimination by the year 2018 [17]. This reduction in malaria burden has largely been due to the key factors of drug and insecticide policy change, strengthening surveillance systems to include active case detection and treatment, cross-border malaria collaboration with neighboring countries and strong political will to ensure that resources were optimized for malaria control post the year 2000.

The Province of KwaZulu-Natal has seen the largest reduction in malaria cases compared to the other two endemic provinces over the 10 years that the data were reviewed. The contributing factors for the observed reduction was due to robust policy changes and collaboration with the malaria project of the LSDI [18]. Moreover, the Province was able to ensure that it attained universal coverage of the key interventions: IRS (>85%), case management (approximately 100% diagnosis using RDT, microscopy and treatment of malaria cases) and a robust, active, case detection program whereby malaria surveillance agents tracked down each malaria case and surveyed neighboring households for parasites [2]. The 380 cases reported in KwaZulu-Natal for 2010 places this Province as a candidate for getting to zero by the target date of 2018.

Funding for the trilateral collaboration on the LSDI has ceased posing a threat to sustaining the gains made through this project. Innovative financing for malaria control is required to continue malaria control efforts in the implementing countries. Approaching private sector partners (especially corporate) may provide a solution.

Mpumalanga Province also recorded significant gains in malaria morbidity and mortality reductions and its strategies and intervention coverage have been similar to that of KwaZulu-Natal. Mpumalanga Province records a large number of imported malaria cases from neighboring Mozambique. The 2010 data show that approximately 70% of the cases arising in Mpumalanga were imported from Mozambique [19]. The health promotion interventions and the surveillance strategy will need to be bolstered to ensure that importation of malaria and secondary transmission is minimized.

The Limpopo Province halved its malaria transmission for the comparison years, however the slow reduction in numbers of deaths is still a cause for concern. Despite the TLMI, reduction in malaria cases in the Limpopo Province compared to the other provinces have not been that marked. This slow reduction of cases could be due to importation of cases and secondary transmission from the neighboring Zimbabwe and Mozambique. Financial resource constraints in Zimbabwe could have contributed to slow progress of implementation of the TLMI, this issue has recently been resolved due Zimbabwe receiving a 2010 Global Fund grant for Malaria.

Mpumalanga Province has also recorded a relatively lower decrease in malaria deaths compared to KwaZulu-Natal. Confidential inquiries into deaths have revealed a combination of factors that were responsible: late presentation of cases and management at health facilities [2]. The strategies for mitigating these challenges will involve ongoing training of health cadres on diagnosis and treatment, and scaling up health promotion for communities to seek treatment early and for South Africa to consider a review of its drug treatment policy to ensure community health workers can diagnose and treat malaria, this issue is currently being addressed by the South African health authorities.

The government of South Africa predominantly provides funding for malaria control interventions with occasional technical support for funding being provided by partners. Financing for malaria is crucial since the gains for malaria control and beyond need to be sustained.

Imported malaria cases (mainly from other endemic countries, but rarely from within South Africa) are increasingly being reported in urban settings such as Gauteng Province. This makes it necessary to undertake studies that seek to understand local and internal migration patterns and its effect on local malaria transmission [19]. Steps to counteract the importation of cases will possibly need to be a combination of interventions including: voluntary screening at borders, enhanced active case detection and treatment and use of health promotion activities. The choice of interventions requires investigation before implementation.

The WHO malaria elimination continuum advocates for determining malaria incidence per thousand population at risk to categorize malaria programs and epidemiological zones into one of four strata: control, pre-elimination, elimination and prevention of reintroduction [20]. South Africa’s malaria incidence per thousand-population at risk in 2010 was 1.14 cases per 1,000 population at risk (approximately 5.9 million) compared to 15.2 per 1,000 population at risk in 2000 (approximately 4.1million) [19]. This places South Africa in the pre-elimination category of the WHO’s malaria elimination continuum. Whilst this is encouraging, for South Africa to get to zero, passive surveillance systems will need to be scaled up to ensure that imported cases are differentiated from local cases, therefore the foci of transmission can be identified and appropriately eliminated.

## Conclusion

South Africa has reached the MDG6 of the malaria target. The country’s achievements are associated with optimal implementation of key interventions: vector control, case management, surveillance, health promotion and robust cross-border malaria control initiatives, such as the LSDI. Additionally, the South African government demonstrated a high level of political commitment where the government dedicated substantive public financial resources to malaria control in the country.

South Africa is now in a position to move beyond the MDG6 targets for malaria and reduce malaria incidence to near zero by 2018. To achieve this, in addition to its existing interventions, South Africa will need to scale up active surveillance coupled with treatment at the community level. Moreover, health systems strengthening, as advocated by others, will be a key component to the malaria program. Programs will especially need to focus on:human resources, information systems infrastructure, transport systems coupled with strengthening and sustaining effective cross-border and health promotion strategies [21,22].

## Abbreviations

ACTs: Artemisinin combination therapy; MDG: Millennium Development Goals; MIS: Malaria Information Systems; LSDI: Lubombo Spatial Development Initiative; RDTs: Rapid Diagnostic Tests; TLMI: Trans-Limpopo Malaria Initiative.

## Competing interests

The authors declare that they have no competing interests.

## Authors’ contributions

DM and RM conceptualized the paper and all other authors provided technical content. All authors read and approved the final manuscript.

## Author details

^1^National Department of Health- South Africa, Civitas Building, Cnr Andries & Struben Street, Pretoria, Gauteng 0001, South Africa. ^2^Harvard School of Public Health, 677 Huntington Avenue, Boston, MA 02115, USA.^3^Clinton Health Access Initiative and Global Health Group, University of California, San Francisco, Pretoria, South Africa. ^4^Limpopo Provincial Government: Department of Health and Social Welfare, PO Box 33, Tzaneen 0850, South Africa. ^5^Mpumalanga Provincial Government: Department of Health and Social Services, Private Bag X11278, Nelspruit, Mpumalanga, South Africa. ^6^KwaZulu-Natal Provincial Government: Department of Health– KwaZulu-Natal, Private Bag X002, Jozini, KwaZulu-Natal, South Africa 3969.^7^Medical Research Council of South Africa, Malaria Research Programme, Medical Research Council, PO Box 70380, Overport, Durban 4067, South Africa.
